# Metagenomics Study of Viral Pathogens in Undiagnosed Respiratory Specimens and Identification of Human Enteroviruses at a Thailand Hospital

**DOI:** 10.4269/ajtmh.16-0062

**Published:** 2016-09-07

**Authors:** Yanfei Zhou, Stefan Fernandez, In-Kyu Yoon, Sriluck Simasathien, Veerachai Watanaveeradej, Yu Yang, Omely A. Marte-Salcedo, Deidra J. Shuck-Lee, Stephen J. Thomas, Jun Hang, Richard G. Jarman

**Affiliations:** ^1^ Viral Diseases Branch, Walter Reed Army Institute of Research, Silver Spring, Maryland; ^2^ Department of Virology, U.S. Armed Forces Research Institute of Medical Sciences, Bangkok, Thailand; ^3^ International Vaccine Institute, Seoul, Republic of Korea; ^4^ Department of Pediatrics, Phramongkutklao Hospital, Bangkok, Thailand

## Abstract

Numerous pathogens cause respiratory infections with similar symptoms. Routine diagnostics detect only a limited number of pathogens, leaving a gap in respiratory illness etiology surveillance. This study evaluated next-generation sequencing for unbiased pathogen identification. Respiratory samples collected in Thailand, Philippines, Bhutan, and Nepal, that were negative by several molecular and immunofluorescence assays, underwent viral cultivation. Samples which demonstrated cytopathic effect in culture (*N* = 121) were extracted and tested by Luminex xTAG respiratory viral panel (RVP) assay and deep sequencing by Roche 454 FLX Titanium system. Using RVP assay, 52 (43%) samples were positive for enterovirus or rhinovirus and another three were positive for respiratory syncytial virus B, parainfluenza 4, and adenovirus. Deep sequencing confirmed the Luminex assay results and identified additional viral pathogens. Human enteroviruses, including *Enterovirus A* type 71 and 12 types of *Enterovirus B* (EV-B) were identified from a hospital in Bangkok. Phylogenetic and recombination analysis showed high correlation of VP1 gene-based phylogeny with genome-wide phylogeny and the frequent genetic exchange among EV-B viruses. The high number and diversity of enteroviruses in the hospital in Bangkok suggests prevalent existence. The metagenomic approach used in our study enabled comprehensive diagnoses of respiratory viruses.

## Introduction

Respiratory tract infections are common human illnesses causing significant morbidity and mortality. Timely clinical diagnostics, constant surveillance, and etiological discovery studies are essential for the control and prevention of infectious diseases.^1^–^3^ One challenge for the control of respiratory diseases is that there are numerous viruses, bacteria, fungi, etc. which can cause acute or incidental illnesses.^4^ The clinical symptoms are often insufficient for making a clear diagnosis without using clinical microbiology and/or molecular tests.^2^ Molecular assays, especially polymerase chain reaction (PCR)–based methods, are routinely used in modern clinical laboratories with speed, sensitivity, and specificity suitable for diagnostic purposes. However, these assays are limited to the designed detection spectrum, having the consequence of missed detection of many pathogens.^5^ Moreover, viruses evolve constantly through mechanisms such as mutagenesis by rapid replication, recombination, or reassortment events. Frequent emergence, reemergence, migration, and even unpredicted introduction of novel or significantly mutated viral pathogens are a considerable challenge to clinical diagnostics which target known pathogens. The robust next-generation sequencing (NGS) technology has the potential to improve underdiagnoses and identify unknown pathogens cost-effectively.^6^,^7^ NGS is advancing toward practical clinical use for sequence-based diagnostics.^8^–^10^

Enteroviruses (genus *Enterovirus*, family *Picornaviridae*, order *Picornavirales*) have 12 species which include hundreds of types.^11^ In the recent revision by the International Committee on Taxonomy of Viruses (http://www.ictvonline.org/), genus *Enterovirus* consists of species *Enterovirus A*-*H*, *Enterovirus J*, and *Rhinovirus A*-*C*. These human and animal viruses can cause various illnesses^12^ with symptoms of varied severity from mild to fatal.^13^ The high prevalence and large diversity of enteroviruses and the outbreaks caused by enteroviruses indicates the need for surveillance and research of *Enterovirus* for better understanding of epidemiology and genomic evolution.

In the present study, we retrospectively investigated 121 clinical respiratory samples that were negative by several molecular tests or immunofluorescence assays (IFAs) for identification of viral pathogens (detailed below) at the Department of Virology, Armed Forces Research Institute of Medical Sciences (AFRIMS), Bangkok, Thailand. Two methods, including Luminex xTAG Respiratory Viral Panel FAST test and Roche 454 GS FLX Titanium pyrosequencing, were used and the results were compared. NGS was applied to unbiased detection of viral sequences in cultured respiratory samples. The outcome was compared with Luminex assays which have received Food and Drug Administration (FDA) clearance for detection of viral pathogens. The acquired viral sequences, especially the whole genome sequences of human enteroviruses, were subjected to comparative analysis to show application of NGS in comprehensive infectious disease surveillance.

## Materials and Methods

### Collection, assay, and viral culture of respiratory specimens.

The Department of Virology, AFRIMS, received respiratory samples collected from influenza-like illness (ILI) surveillance conducted at U.S. Embassy Medical Units in south and southeast Asia and Phramongkutklao (PMK) Hospital in Bangkok, Thailand, from 2008 to 2011 (Supplemental Table 1). The surveillance activities were performed for public health purposes or were undertaken under protocols approved by the institutional review boards of the Walter Reed Army Institute of Research (WRAIR) and the host country institution. Nasopharyngeal swabs were collected from patients with signs of ILI and stored in universal transport media (Copan Diagnostics, Murrieta, CA). Respiratory samples were tested at AFRIMS for influenza A viruses (H1, H3, H5, and N1 genes) and influenza B viruses by the U.S. Centers for Disease Control and Prevention 5-plex PCR and/or IFA on cultured Vero, LLC-MK2, and H292 cells, to identify influenza A and B, human adenoviruses, respiratory syncytial virus (RSV), and parainfluenza viruses (PIVs). For respiratory samples that were negative in these tests, but with apparent cytopathic effect (CPE), viral cultures of 1–3 passages were stored at −80°C and shipped to the Viral Disease Branch, WRAIR, for further investigation (Supplemental Table 1).

### Extraction of nucleic acids.

QIAamp viral RNA mini kit (Qiagen, Valencia, CA) was used in the purification of nucleic acids without DNase I treatment step from clear supernatants of viral cultures. Carrier RNA (Qiagen) was used to protect RNA sample from degradation. Bacteriophage MS-2 RNA (Roche Diagnostics Corporation, Indianapolis, IN) was added as an internal control.

### Luminex respiratory viral panel assay.

Luminex xTAG respiratory viral panel (RVP) assay and instrument Luminex 200 (Luminex, Austin, TX) were used to test the nucleic acid samples for detection of viral pathogens and subtypes, including influenza A, influenza A subtype H1, influenza A subtype H3, influenza B, PIVs, RSVs, human metapneumovirus, enteroviruses and rhinoviruses, and adenoviruses.

### Random nucleic acid amplification and pyrosequencing.

The procedure has been described in detail previously.^14^ In brief, the nucleic acids were subjected to random amplifications including reverse transcription with anchored random primers and PCR with anchored random primers and the primer specific to the anchor sequence. To sequence the random amplicons, GS FLX Titanium Rapid Library Preparation Kit (Roche 454 Life Sciences, Branford, CT) was used to make barcoded libraries. After purification, the DNA size distribution of the RL library was examined by PCR assay. There was no size selection of the library performed, unless there was a large quantity of small fragments (i.e., ≤ 100 base pairs [bp]) in the PCR assay results. In size selection to remove the short fragments, the RL library was resolved on a 2% agarose gel; the smeared band with the size ≥ 150 bp was collected and subjected to DNA gel extraction. The RL library copy number concentration was determined using a RL standard and a QuantiFluor-P fluorometer (Promega, Madison, WI). Subsequent emulsion PCR and pyrosequencing on the GS FLX system (Roche 454 Life Sciences) were performed by following standard protocols, except that PCR primer concentration was reduced by 75% in amplicon emulsion. Subsequent sequencing was performed with the Roche/454 GS FLX Titanium system (Roche 454 Life Sciences).

### Sequence-based pathogen identification.

Pyrosequencing data were subjected to demultiplexing, removal of primer sequence,^14^ trimming off terminal bases with low quality score, and removal of quality processed reads with length shorter than 50 bases. Roche GS FLX software GSAssembler version 2.3 was used in the de novo assembly of pyrosequencing reads. Assembly contigs and the unassembled reads were subjected to BLASTN search against nucleotide sequence databases for viruses and bacteria from GenBank database (ftp://ftp.ncbi.nlm.nih.gov/blast/db/). The unidentified sequences were subjected to megablast with GenBank nucleotide collection (nt/nr) database and BLASTX against nonredundant protein sequences (nr). For human enterovirus sequences, an online genotyping tool (http://www.rivm.nl/mpf/enterovirus/typingtool#/) was used to determine type enteroviruses based on capsid protein VP1 gene sequences.^15^

### Phylogenetic and recombination analysis.

Sequence data analysis software Geneious Pro version v 5.6.4 (Biomatters, Auckland, New Zealand) and Molecular Evolutionary Genetics Analysis version 6.0 (MEGA6)^16^ were used for nucleotide sequences alignment, data manipulation, and phylogenetic analyses. Neighbor-joining and maximum likelihood methods were used in the construction of the phylogenetic tree and confirmation of phylogenetic analysis results. Software Recombination Detection Program (RDP) version 4.38 was used to characterize viral genome recombination events.^17^ Complete genome sequences of prototype strains (listed in http://www.picornaviridae.com/) and related isolates were used as reference in the analysis of human enteroviruses identified in this study.

## Results

**Figure 1.**
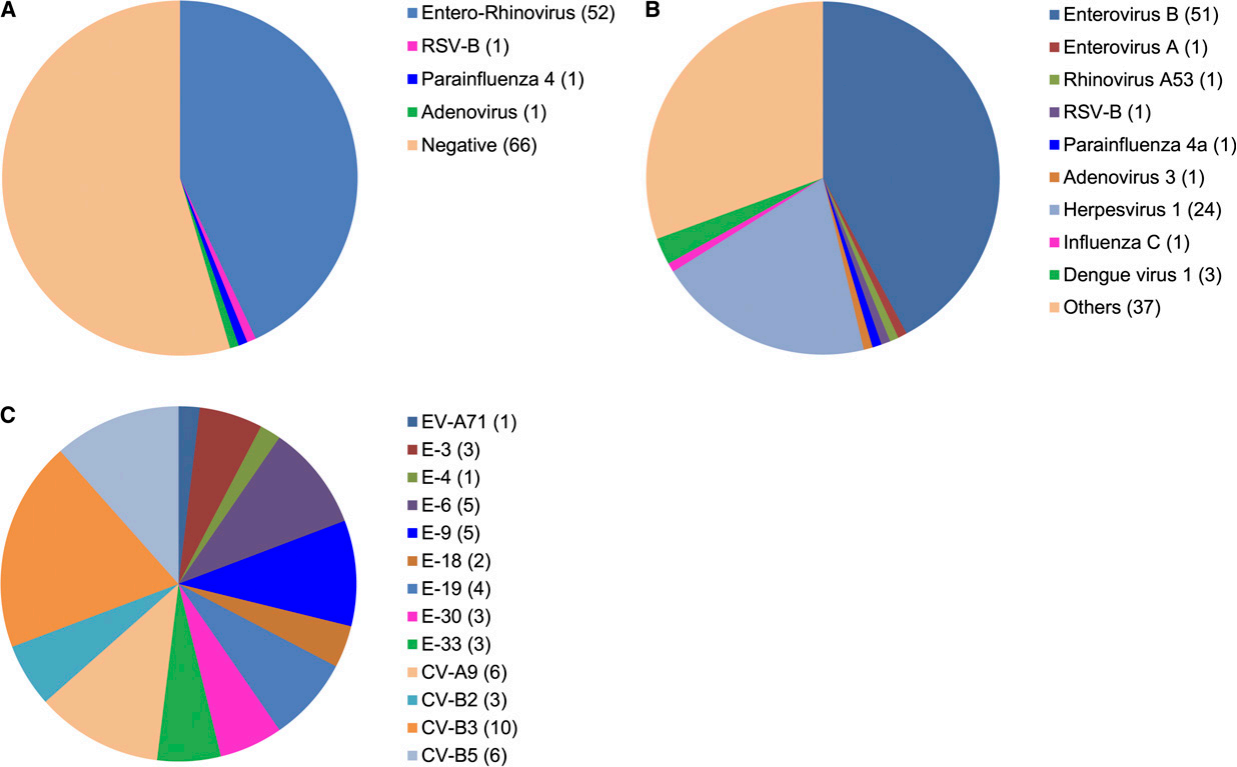
Identification of viral pathogens by using (**A**) Luminex xTAG Respiratory Viral Panel FAST test or (**B**) Roche 454 GS FLX Titanium pyrosequencing. (**C**) Genotypes of enteroviruses determined with assembled sequences. Numbers in parentheses are the numbers of samples containing the virus, species, or genotype; negative in test or containing other nonviral pathogens.

We studied 121 CPE-positive respiratory samples by viral culture, Luminex RVP assay, and GS 454 pyrosequencing to find potential causative agents which had not been identified in previous conventional PCR and IFA tests (detailed in Material and Methods). The original specimens were collected from Thailand (*N* = 89), Philippines (*N* = 22), Bhutan (*N* = 4), and Nepal (*N* = 6). The clinical data and the Luminex RVP and sequencing results are summarized in Table 1 and Figure 1

; the detailed records are shown in Supplemental Table 1. Moreover, phylogeny and recombination analysis were performed for the human enteroviruses identified in the study.

### Identification of additional viral pathogens.

Additional testing at WRAIR identified a variety of other viral pathogens in these cultured respiratory samples: 55 of 121 (45.5%) were positive in Luminex RVP assay (Figure 1A), whereas viral pathogen sequences were found in 84 of 121 samples (69.4%) (Figure 1B). All viruses detected by the Luminex assay were also identified by random pyrosequencing. In the present study, approximately 5,000 or more 454 reads were generated for each sample for the analysis. The sequences also allowed genotyping of all these viruses. In addition, more viruses were identified using the unbiased metagenome sequencing approach than detected in RVP assay. Human type 1 herpesviruses, which are frequently present in the respiratory tract, were seen in a number of samples (*N* = 24). Other identified pathogens included influenza C virus (*N* = 1) which is usually not tested for, and dengue virus type 1 (*N* = 3) which is an arbovirus that can cause some respiratory symptoms, and has been previously detected and isolated from nasal and throat swabs.^18^

### Determination of types of the human enteroviruses.

The majority of the identified viral pathogens, 52 of 84 (61.9%), belonged to human enteroviruses. All samples were from PMK Hospital in Bangkok, Thailand, except for sample EBMSV0003 which was from the U.S. Embassy in Bangkok. All but three patients (18, 23, and 47 years of age) were children. Two patients (3 and 2 years of age) were hospitalized for 3 days and 3 weeks, respectively. These observations are consistent with a previous report on pan-enterovirus epidemic of hand, foot, and mouth diseases (HFMD) and herpangina in Thailand in 2008–2011.^19^ Of the 52 enteroviruses we identified, only one sample, PMKA1300, contained *Enterovirus A* type 71 (EV-A71) virus; all others were *Enterovirus B* (EV-B) viruses.

Whole genome sequences of all 52 enteroviruses were assembled and used to type the viruses with the online genotyping tool developed by Kroneman and others.^15^ The genotyping was based on phylogeny of VP1 gene and done individually. A total of 81 VP1 genes chosen as reference in the typing were downloaded, and together with the 52 enterovirus sequences, were aligned, adjusted, and used in construction of the phylogenetic tree to show all reference strains and the samples in the present study. VP1-based genotyping showed the 52 enteroviruses from Bangkok belonged to EV-A71 subgenogroup B5 (*N* = 1), which caused epidemics and large outbreaks in multiple Asian areas,^20^ and 12 different types of EV-B viruses (*N* = 51) (Figure 1C).

### Genome-wide phylogenetic analysis of EV-B sequences.

Most whole genome studies of enteroviruses endemic in Asian countries were for EV-A71 viruses which are associated with severe cases more often than other types.^21^,^22^ As a consequence, genomes of EV-B, the most diverse enterovirus species have not been well investigated. In the present study, we obtained complete coding sequences for 51 EV-B viruses of multiple genotypes. First, we aligned all 52 whole genome sequences, which consisted of complete coding sequences and most terminal sequences, then analyzed the phylogenetic relationships among them (Supplemental Figure 1). The genome-wide and VP1-based phylogenies were found to be consistent without discrepant placement of any one virus in the phylogenetic tree clusters. The observation provided strong evidence that VP1 genes have similar evolutionary traits as the whole genomes of EV-B viruses indicating that VP1 genes may be useful in genotying of enteroviruses.

**Figure 2.**
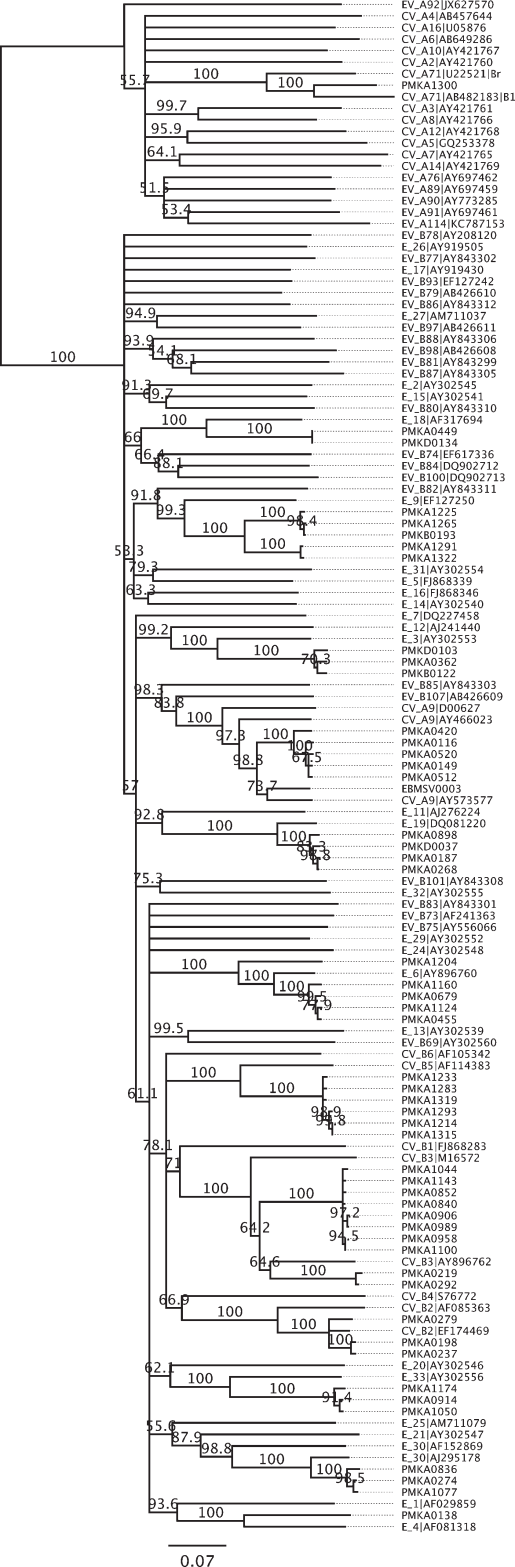
Enterovirus genotyping for the Phramongkutklao (PMK) samples by neighbor-joining phylogenetic analysis of nucleotide sequences for capsid protein VP1 genes with 1,000 bootstrap replications. GenBank accession number for each reference sequence is shown after the type name. The bar indicates the pairwise sequence distance of average nucleotide substitutions per site.

The EV-B genomes were further characterized by whole-genome phylogenetic analysis. The analysis included genome sequences of 36 EV-B prototype strains listed in Picornavirus.com, EV-B sequences from a study by Oberste and others,^23^ 13 recent EV-B sequences in GenBank, and the 21 EV-B genome types from this study which had a nucleotide divergence of 0.03 or greater among each other (Supplemental Figure 1). Phylogenetic analysis using nucleotide sequences of these 70 EV-B genomes failed to produce a stable tree of evolutionary lineage between these highly divergent viruses. Instead, complete amino acid sequences were used in the analysis and revealed EV-B phylogeny to be consistent with that based on VP1 genes. The result shows the high sequence divergence among types of EV-B viruses, and the divergence of varying distances between the prototypes with more recent strains and those from this study (Figure 2

).

### Genetic recombination analysis of EV-B viruses.

**Figure 3.**
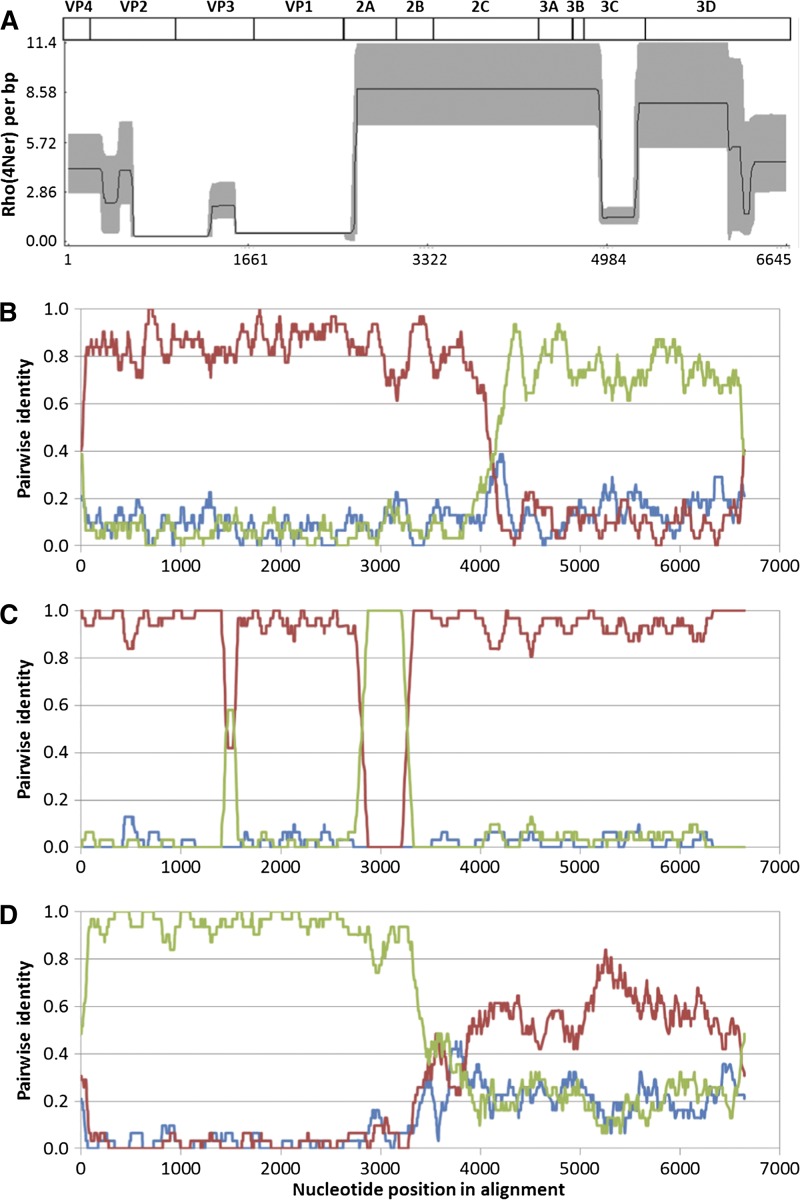
Detection of recombination among 21 *Enterovirus B* (EV-B) virus genotypes in this study. Complete coding sequences were aligned by MUSCLE,^16^ and then analyzed with software package Recombination Detection Program.^17^ (**A**) Recombinant rate plot by LDHat analysis of all 21 genotypes, with block penalty setting of 10 and Monte Carlo Markov Chain updates setting of 1,000,000.^17^ Mature proteins and gene locations are shown above. The solid line indicates the site-by-site recombination rate estimate. The gray zone indicates 95% credibility interval. (**B**–**D**) Possible recombination events in **B**. PMKA0279. The graph shows pairwise alignment identity of PMKA0279 vs. PMKA0198 (red line), PMKA0279 vs. PMKA0836 (green line), and PMKA0836 vs. PMKA0198 (blue line). (**C**) PMKA1174. The graph shows pairwise alignment identity of PMKA1174 vs. PMKA0914 (red line), PMKA1174 vs. PMKA0134 (green line), and PMKA0914 vs. PMKA0134 (blue line). (**D**) PMKA0836. The graph shows pairwise alignment identity of PMKA0836 vs. PMKA0362 (red line), PMKA0836 vs. PMKA0274 (green line), and PMKA0362 vs. PMKA0274 (blue line).

**Figure 4.**
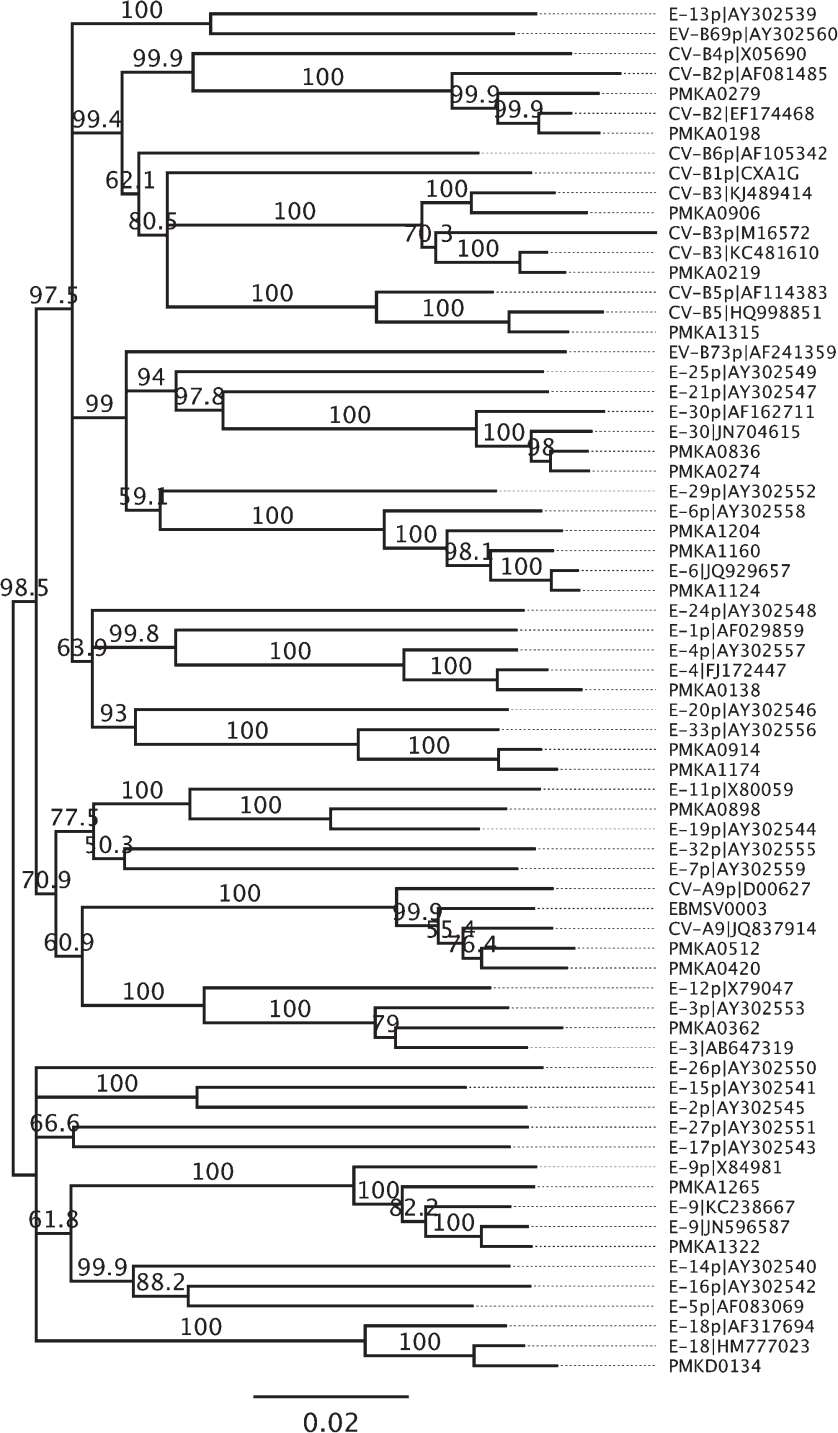
Phylogeny of *Enterovirus B* (EV-B) viruses from this study and reference strains. Polyprotein amino acid sequences were aligned and used in neighbor-joining phylogenetic analysis with 1,000 bootstrap replications. GenBank accession numbers for reference strains were shown. The bar indicates the pairwise sequence distance of average amino acid substitutions per site.

We identified 51 EV-B viruses which were phylogenetically classified into 12 types and 21 different genotypes. It is intriguing to explore the possible recombination events between these genotypes coexisting around 2010–2011 in Bangkok. RDP analysis of complete protein-coding sequences indicated multiple probable recombination patterns among the 21 genotypes. The recombination rate plot by LDHat^24^ (Figure 3A

) interestingly showed that the enterovirus capsid proteins, which are encoded by sequences 1A-1D that are much more variable than other regions (2A-3D), have lower levels of recombination. In particular, the entire VP1 gene locates within the region with the lowest frequency of recombination. This observation is in agreement with the finding that VP1-based phylogeny was consistent with genome-wide phylogenetic analysis (Figures 2 and 4

). RDP analysis suggested that a variety of recombination arrangements may exist for genetic exchanges among enteroviruses. PMKA0279 (CV-B2) is likely a recombinant of two parental viruses homologous with PMKA0198 (CV-B2) and PMKA0836 (E-30); whereas these two parental viruses are unrelated (Figure 3B). PMKA1174 (type E-33) is highly similar with PMKA0914 (E-33) with an approximate 450-nucleotide region transferred from PMKA0134 (E-18) (Figure 3C). PMKA0836 (type E-30) has its 5′ half of CDS very close to PMKA0274 (E-30) with nucleotide identity of 97.5%, whereas the parental sequence for its 3′ half was not found in the viruses in the present study, but may be related to PMKA0362 (E-3) (Figure 3D).

### Nucleotide sequence accession numbers.

The genome sequences containing complete polyprotein gene sequences for EV-A71 B5 virus PMKA1300 and 21 EV-B viruses representing 12 different types were deposited in GenBank under accession numbers KU574619 to KU574640.

## Discussion

Numerous molecular assays have been routinely used in clinical diagnostics with increasingly improved speed, sensitivity, and accuracy. The latest NGS technology has become more clinically relevant, and its utility has been documented in cases where standard clinical tests did not yield a diagnosis.^8^,^25^–^27^ In this retrospective study, we investigated 121 viral cultured clinical specimens by using the FDA-cleared Luminex xTAG RVP assay and NGS random sequencing. The study evaluated prescreened CPE-positive samples that were negative in initial assays of influenza surveillance (Supplemental Table 1). We focused on comparison of the Luminex and NGS approaches and identification of pathogens which were not typically monitored in hospitals. As expected, all viruses that were positive by Luminex RVP were also detected by NGS. Moreover, NGS allowed genotyping of the viruses and the identification of other respiratory viruses and viruses such as Dengue virus which are not considered as respiratory pathogens. In samples with no detected viruses, other bacterial sequences such as *Clostridium difficile*, *Acinetobacter baumannii*, *Streptococcus mitis*, or *Streptococcus pneumonia* were found, suggestive of possible nonviral infections or colonization (data not shown). It is noted that viral cultivation of the specimens was done initially for IFA, and the supernatants were then extracted and used in the present study. For the NGS-based method, viral culture of clinical samples effectively enriched viral contents, if the virus was viable and could propagate, resulting in more viral sequence reads and assembly of viral genome sequences. On the other hand, for viruses less abundant or did not grow as well as bacterial pathogens, NGS in this study was still sensitive enough to identify them with individual reads. However, including a viral cultivation step may not be viable when taking account of the associated costs of time, efforts and expenses, and the risk of contamination.

The use of culture, advanced molecular assays, and the unbiased deep sequencing in analyzing fresh or archived clinical samples has led to more comprehensive understanding of the prevalence of viruses as well as bacteria and other microbes in clinical environments. With the findings of multiple viruses and increased incidence of mixed infections, enhanced data analysis to integrate information of multiple aspects is critical for accurate interpretation. More evidence will be needed to determine whether enteroviruses and other viruses found in this study were the primary causes of the symptoms.

The majority (52/84) of viruses identified in the present study were enteroviruses, especially EV-B viruses, indicating the possible high prevalence of enterovirus among respiratory infections as suggested in a study of non-rhinovirus enteroviruses in Peru^28^ and in a review of acute exacerbations of chronic obstructive pulmonary disease.^29^ Our study produced a large set of genome sequences which contain complete coding sequences for EV-A71-B5 and EV-B of diverse genotypes in Thailand, whereas most previous genome studies in Asia focused on HFMD pathogen EV-A71.^21^,^30^ Phylogenetic and recombination analysis in the present study suggest VP1 gene is consistent with genome or CDS sequence in phylogeny-based typing of enteroviruses, whereas the more conserved protease and polymerase sequences are not suitable for genotyping due to the high recombination rate in these regions. Other studies showed the 5′-untranslated region may also have frequent recombination events and can cause misdiagnosis if used in genotyping.^31^,^32^ The VP1 gene, in spite of its high sequence variability,^23^ remains the most suitable for sequence-based enterovirus (EV) typing.^32^–^34^ An alternative sequence, the VP4/VP2 region was used in several studies.^31^ Our analysis suggests the VP2/VP3 region might be a reasonable alternative in EV typing, if needed. More work can be done to validate and compare its usefulness.

## Supplementary Material

Supplemental Datas.

## Figures and Tables

**Table 1:** Summary of clinical data of patients in this study

Group (ages)	Toddlers (0–3)	Children (3–12)	Teens (12–19)	Adults (> 19)	No record	Total
No. of patients (%)	*N* = 46 (38.0)	*N* = 50 (41.0)	*N* = 7 (5.8)	*N* = 16 (13.2)	*N* = 2 (1.7)	*N* = 121 (100)
Sex	(0)*	(0)	(0)	(0)	(2)	(2)
Female	18/46 (39.1)†	24/50 (48.0)	2/7 (28.6)	3/16 (18.8)	0	47/119 (39.5)
Male	28/46 (60.1)	26/50 (52.0)	5/7 (71.4)	13/16 (81.2)	0	72/119 (60.5)
Severe cases						
Hospitalized	4/46 (8.7)	2/50 (4.0)	1/7 (14.3)	1/16 (6.3)	0	8/121 (6.6)
High temperature (> 39°C)	5/46 (10.9)	5/50 (10.0)	0/7 (0.0)	2/16 (12.5)	0	12/121 (9.9)
Cough	(0)	(1)	(1)	(3)	(2)	(6)
Yes	44/46 (95.7)	43/49 (87.8)	5/6 (83.3)	8/13 (61.5)	0	100/114 (87.7)
No	2/46 (4.3)	6/49 (12.2)	1/6 (16.7)	5/13 (38.5)	0	14/114 (12.3)
Sore throat	(14)	(2)	(1)	(3)	(1)	(21)
Yes	21/32 (65.6)	30/48 (62.5)	4/6 (66.7)	6/13 (46.2)	1	62/100 (62.0)
No	11/32 (34.4)	18/48 (37.5)	2/6 (33.3)	7/13 (53.8)	0	38/100 (38.0)
Running nose	(13)	(12)	(2)	(9)	(2)	(40)
Yes	27/31 (87.1)	31/38 (81.5)	3/5 (60.0)	3/7 (42.9)	0	64/81 (79.0)
No	4/31 (12.9)	7/38 (18.4)	2/5 (40.0)	4/7 (57.1)	0	17/81 (21.0)
Headache	(22)	(4)	(0)	(3)	(1)	(37)
Yes	6/19 (31.6)	17/44 (38.6)	7/7 (100)	9/13 (69.2)	1	40/84 (47.6)
No	13/19 (68.4)	27/44 (61.4)	0/7 (0.0)	4/13 (30.8)	0	44/84 (52.4)
Chill	(11)	(5)	(1)	(3)	(2)	(22)
Yes	9/35 (25.7)	9/45 (20.0)	1/6 (16.7)	10/13 (76.9)	0	29/99 (29.3)
No	26/35 (74.3)	36/45 (80.0)	5/6 (83.3)	3/13 (23.1)	0	70/99 (70.3)

* Number in parentheses is the number of patients with no record of the information.

† The result is shown as number of patients/total number of patients with known information and the percentage in parentheses.
