# Supplementary material for: Metagenomics Study of Viral Pathogens in Undiagnosed Respiratory Specimens and Identification of Human Enteroviruses at a Thailand Hospital
Source: Am J Trop Med Hyg. 2016 Sep 7;95(3):663–9. doi: 10.4269/ajtmh.16-0062 (PMC5014275; doi:10.4269/ajtmh.16-0062)
Supplement: Supplementary file 1 [file SD6.pdf]

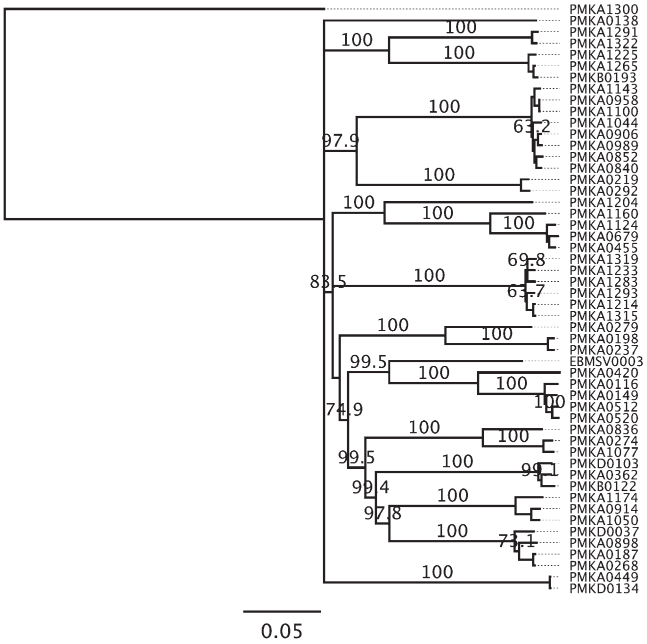

Supplemental Figure 1. Evolutionary distances among the 52 enteroviruses estimated by genome-wide phylogenetic analysis. Genome sequences were aligned by MUSCLE and used in construction of phylogenetic tree by neighbor-joining method with Jukes–Cantor genetic distance model and 1,000 bootstrap replications.
